# Differences in left ventricular functional adaptation to arterial stiffness and neurohormonal activation in patients with hypertension: a study with two-dimensional layer-specific speckle tracking echocardiography

**DOI:** 10.1186/s40885-017-0078-9

**Published:** 2017-11-02

**Authors:** Darae Kim, Chi Young Shim, Geu-Ru Hong, Sungha Park, InJeong Cho, Hyuk-Jae Chang, Jong-Won Ha, Namsik Chung

**Affiliations:** Cardiology Division, Severance Cardiovascular Hospital, Yonsei University College of Medicine, 50 Yonsei-ro, Seodaemun-gu, 120-752 Seoul, Republic of Korea

**Keywords:** Left ventricle, Arterial stiffness, Aldosterone

## Abstract

**Background:**

Arterial stiffness increases pressure load to the left ventricle (LV), leading to LV hypertrophy and subendocardial ischemia. Neurohormones stimulate myocardial fibrosis and LV dysfunction. We aimed to explore the associations of arterial stiffness and plasma aldosterone with multi-directional, layer-specific LV, and left atrial (LA) mechanical function in hypertensive patients.

**Methods:**

Layer-specific LV global longitudinal strain (GLS-trans, GLS-endo, GLS-epi), global circumferential strain (GCS-trans, GCS-endo, GCS-epi), LV torsional parameters, and LA global longitudinal strain (LA GLS) were analyzed by two-dimensional speckle tracking echocardiography in 195 hypertensive patients (110 men, mean age 55 years). Pulse wave velocity (PWV) was analyzed as a measure of arterial stiffness, and plasma aldosterone was measured for evaluation of neurohormonal activation.

**Results:**

In a simple correlation, PWV significantly correlated with LV GLS-endo and LA GLS. Log aldosterone correlated with both LV GCS-endo and LV GCS-trans. Multiple regression analysis revealed that LV GLS-endo (β = 0.223, *p* = 0.031) and LA GLS (β = −0.311, *p* = 0.002) were independently correlated with PWV even after controlling for confounding factors.

**Conclusions:**

In hypertensive patients without clinically apparent target organ damage, LV GLS, especially endocardium, and LA GLS were more dominantly affected by arterial stiffness because, among the three myocardial layers, the endocardium is most susceptible to pressure overload. Two-dimensional layer-specific speckle-tracking echocardiography sensitively detects LV mechanical dysfunction and provides pathophysiologic insights into LV mechanical adaptations in hypertension.

## Background

Cardiac remodeling in hypertensive disease is a complex process involving both mechanical stress from central arterial stiffness and neurohormonal factors [1]. Arterial stiffness increases pressure load to the LV, increasing the size of myocytes and leading to LV hypertrophy. Neurohormones also play important roles in adverse remodeling of LV. Previous experimental studies revealed that aldosterone induced myocardial fibrosis, hypertrophy, and dysfunction [2, 3].

Progressive remodeling of LV is suggested to be heterogeneous across the LV wall [4]. The myocardium is composed of three myocardial layers; during the course of LV remodeling, central arterial stiffness and neurohormones can have different degrees of impact on the layers of the myocardium. Two-dimensional speckle tracking echocardiography provides quantitative information regarding LV deformation by longitudinal, circumferential, and torsion strain of myocardium [5]. Recently, layer-specific speckle-tracking echocardiography was reported to allow quantification of LV global longitudinal strain (GLS) and circumferential strain (GCS) of the three layers of the myocardium [6].

The aim of this study was to explore the associations of central arterial stiffness and plasma aldosterone with layer-specific LV and LA mechanical function in patients with early hypertension using two-dimensional speckle tracking echocardiography.

## Methods

### Study subjects

This study was designed as a retrospective cohort study. We included consecutive adult hypertensive patients without apparent end organ damage who underwent clinically indicated echocardiography between 2010 and 2015. Patients with reduced ejection fraction (< 50%), significant valvular heart disease, ischemic heart disease, idiopathic dilated cardiomyopathy, restrictive cardiomyopathy or hypertrophic cardiomyopathy, or atrial fibrillation were excluded from the study. Finally, a total of 195 patients were reviewed. Hypertension (HTN) was defined as systolic blood pressure (SBP) ≥ 140 mmHg, diastolic blood pressure (DBP) ≥ 90 mmHg, or current pharmacological treatment for HTN. Clinical data at the time of echocardiography were gathered from electronic medical records. Venous blood was collected in the morning after a 12-h fast with the patients in the sitting position to measure plasma aldosterone and renin on the same day as the echocardiography. Plasma aldosterone concentration (ng/dL) was measured by radioimmunoassay (Diagnostic Systems Laboratories Inc., TX). Plasma renin activity was measured by radioimmunoassay of angiotensin I in the presence of reagents that inhibit angiotensin I–converting enzyme and angiotensinases. The assay was performed according to the method of Sealey using gammacoat plasma renin activity radioimmunoassay kits (DiaSorin [Stillwater, MN], lower limit of determination is 0.1 ng/mL/h).

### Assessments of peripheral and central BPs

Peripheral SBP and DBP measurements were performed automatically (Omron M4 Plus, Japan) at the brachial artery of the non-dominant arm in a relaxed seated position. Two BP measurements obtained at an interval of 5 min during the same visit were averaged. Central hemodynamics and parameters were assessed with pulse wave analysis of the radial artery using commercially available radial artery tonometry (SphygmoCor, AtCor Medical, Sydney, Australia) [7, 8]. The measurements were obtained in the supine position after a minimum of 5 min of rest just before the echocardiogram. Peripheral pressure wave form was recorded from the radial artery with a high-fidelity micromanometer (Millar Instruments, Houston, TX) [8, 9]. Central systolic blood pressure (BP), diastolic BP, pulse pressure (PP), augmentation pressure, and augmentation index (AIx) were analyzed from 20 sequential pulse waveforms. PP was calculated as the difference between SBP and DBP. Augmentation pressure was the maximum systolic pressure minus pressure at the inflection point. The AIx was defined as AP divided by PP and expressed as a percentage. As in previous studies, because AIx is influenced by the heart rate, it was normalized for a heart rate of 75 bpm (AIx@75) [10]. Pulse wave velocity (PWV) was measured using carotid-femoral pressure pulse transit time.

### Two-dimensional and Doppler echocardiography

Each patient underwent a comprehensive transthoracic echocardiographic study using a Vivid 7 or Vivid 9 cardiovascular ultrasound system (GE Medical Systems, Horten, Norway), equipped with 2.5–3.5 MHz phased-array sector probes. Standard 2D and Doppler measurements were performed according to the recommendations of the American Society of Echocardiography guidelines [11]. LV hypertrophy was diagnosed according to the American Society of Echocardiography recommended formula for estimation of LV mass index and was indexed to body surface area (cutoff values for LV mass index were > 115 g/m^2^ for men and > 95 g/m^2^ for women). From the apical window, mitral inflow velocities were traced, and the following variables were obtained: peak velocity of early diastolic mitral inflow (E), late diastolic mitral inflow (A), and deceleration time of the E velocity. Early diastolic mitral annular velocity (e’), late diastolic mitral annular velocity, and systolic mitral annular velocity (S′) were measured from the apical four-chamber view with a 2- to 5-mm sample volume placed at the septal corner of the mitral annulus.

### Two-dimensional speckle tracking strain analysis

Three consecutive cardiac cycles were recorded and averaged, and the frame rates were set to 60–80 frames/s. The analysis was performed offline using customized software (EchoPAC PC, version 113; GE Medical Systems). The endocardial border of the LV was manually traced from three apical views (apical 4-, 2-, and 3-chamber views) to obtain LV global longitudinal strain of endocardial (GLS-endo), transmural (GLS-trans), and epicardial strains (GLS-epi) by averaging all regional peak longitudinal strains. Layer-specific LV global circumferential strain (GCS) was obtained from averaging all circumferential strains from the short axis view of the mitral valve, papillary muscle, and LV apex level (LV GCS-endo, LV GCS-trans, LV GCS-epi). LV Torsion was automatically calculated as the instantaneous difference between LV apical and LV basal rotation.

The global longitudinal strain of the LA (LA-GLS) was measured by manually tracing the LA endocardial border in both four-chamber and two-chamber views. Because two segments of the LA roof demonstrated lower longitudinal strain curves than the other four, they were excluded from both 4-chamber and 2-chamber views. Therefore, global peak LALS during ventricular systole was measured by averaging the values obtained in the eight other LA segments. The time to peak LALS was also measured as the average of the eight segments and by calculating the time delay from the QRS to the positive peak LALS [12].

To examine intraobserver and interobserver variability for layer-specific LV GLS and GCS, the same observer and a second independent observer repeated the analysis of the first 10 consecutive patients. Intraobserver and interobserver intraclass coefficients were as follows: 0.97 (95% confidence interval [CI] 0.96–0.98) and 0.96 (95% CI 0.96–0.98) for LV GLS-endo; 0.96 (95% CI 0.93–0.98) and 0.95 (95% CI 0.90–0.99) for LV GLS-trans; 0.93 (95% CI 0.78–0.96) and 0.89 (95% CI 0.64–0.95) for LV GLS epi; 0.93 (95% CI 0.90–0.98) and 0.95 (95% CI 0.92–0.98) for LV GCS-endo; 0.96 (95% CI 0.93–0.98) and 0.94 (95% CI 0.89–0.98) for LV GCS-trans; 0.91 (95% CI 0.75–0.96) and 0.88 (95% CI 0.62–0.95) for LV GCS epi; 0.88 (95% CI 0.84–0.92) and 0.90 (95% CI 0.85–0.95) for LA GLS.

### Statistical analysis

Continuous variables are presented as mean ± SD and categorical variables are presented as absolute and relative frequencies (%). Spearman’s simple correlation analyses were performed to determine the associations between strain parameters and parameters of arterial stiffness and log aldosterone. Multiple linear regressions were performed to determine the independent association of LV mechanical parameters with arterial stiffness and neurohormone.

## Results

### Demographic characteristics

Table 1 shows baseline characteristics of our cohort. Mean age was 55 ± 13, and 96 patients (49%) were men. Regarding medication, 108 (55%) patients were taking angiotensin-converting enzyme inhibitors or angiotensin receptor blockers at the time of echocardiography. LV hypertrophy was present in 52 (27%) patients. Table 2 shows echocardiographic findings in our cohort. Mean left atrial volume index (24.5 ml/m^2^ ± 7.9) and E/E’ value (10.4 ± 3.5) were in the normal range. The mean value of LV GLS-trans was −16.9 ± 2.9, which was slightly lower than the normal value [11].

**Table 1:** Baseline characteristics of the study population

	*n* = 195
Demographics	
Age, years	55 ± 13
Male gender, n (%)	96 (49)
Body mass index, kg/m^2^	24.8 ± 3.7
Dyslipidemia, n (%)	50 (26)
Smoking, n (%)	21 (11)
Medications	
RAAS blockers, n (%)	108 (55)
Beta blockers, n (%)	39 (20)
Calcium channel blockers, n (%)	68 (35)
Diuretics, n (%)	19 (10)
Hemodynamics	
Peripheral SBP, mmHg	148 ± 76
Peripheral DBP, mmHg	83 ± 13
Peripheral PP, mmHg	61 ± 15
Central SBP, mmHg	132 ± 18
Central DBP, mmHg	85 ± 13
Central PP, mmHg	48 ± 14
AIx@75, %	24 ± 12
Heart rate, bpm	68 ± 12
PP amplification	1.31 ± 0.21
PWV, m/s	8.3 ± 1.8
Aldosterone, ng/dL	16.03 (12.15–19.53)
Renin, ng/mL/h	1.55 (0.68–3.71)

*RAAS* renin angiotensin aldosterone system, *SBP* systolic blood pressure, *DBP* diastolic blood pressure, *PP* pulse pressure, *AIx* augmentation index, *PWV* pulse wave velocity

**Table 2:** Two-dimensional, Doppler, and speckle tracking echocardiography

	*n* = 195
Two-dimensional and Doppler	
LV end-diastolic dimension, mm	48 ± 5
LV end-systolic dimension, mm	31 ± 4
IVS thickness, mm	9.6 ± 1.9
PW thickness, mm	9.4 ± 1.4
Relative wall thickness	0.41 ± 0.25
LV mass index, g/m^2^	92 ± 25
LV hypertrophy, n (%)	52 (27)
LV ejection fraction, %	67.9 ± 5.8
e’ velocity, cm/s	6.8 ± 2.5
S′ velocity, cm/s	7.4 ± 1.5
E/e’	10.4 ± 3.5
LA volume index, ml/m^2^	24.7 ± 7 .9
Two-dimensional speckle tracking	
LV GLS, %	
Transmural	−16.9 ± 2.9
Endocardial	−19.1 ± 3.3
Epicardial	−14.9 ± 2.8
LV GRS, %	34.1 ± 12.2
LV GCS, %	
Transmural	−22.0 ± 5.0
Endocardial	- 34.3 ± 6.9
Epicardial	−15.4 ± 5.7
LV Torsion, peak (°)	27.2 ± 9.5
LA GLS, %	31.7 ± 9.1

*LV* left ventricle, *IVS* interventricular septum, *PW* posterior wall, *LA* left atrium, *GLS* global longitudinal strain, *GRS* global radial strain, *GCS* global circumferential strain, *SD* standard deviation

### Central hemodynamics and neurohormone and the indices of LV mechanical function

**Fig. 1:**
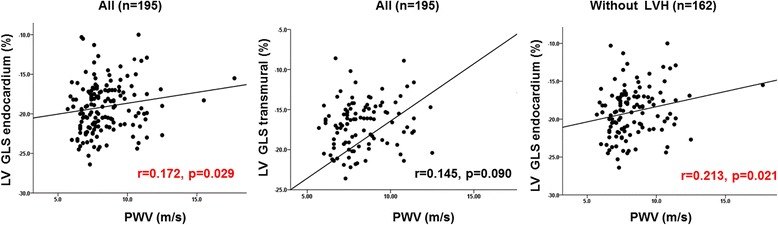
Correlation between strain parameters and PWV

**Fig. 2:**
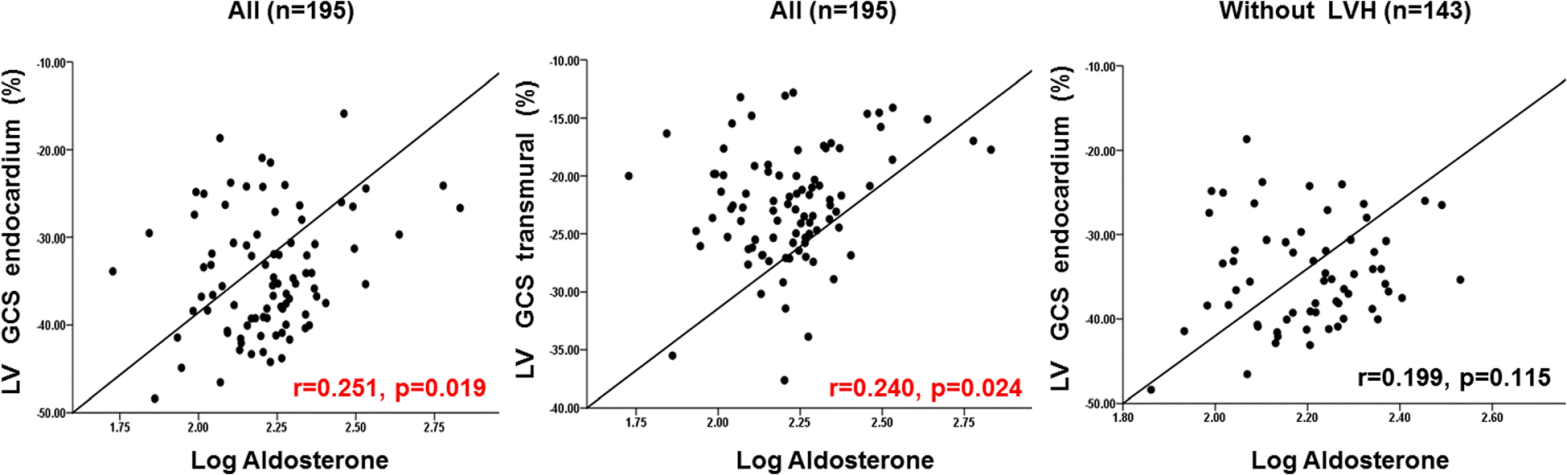
Correlation between strain parameters and log aldosterone

Table 3 shows simple correlation analysis between strain parameters and PWV or log aldosterone. In a simple correlation, PWV was well correlated with LV GLS-endo (*r* = 0.169, *p* = 0.046) and LA GLS (*r* = −0.250, *p* = 0.003). PWV showed significant correlation with LV GLS-endo even in patients without LV hypertrophy(Fig. 1). Other central hemodynamic parameters, such as PP amplification or AIx@ 75, did not show significant correlation with strain parameters. Log aldosterone showed positive correlation with LV GCS-endo (*r* = 0.251, *p* = 0.019) and LV GCS-trans (*r* = 0.240, *p* = 0.024), whereas no significant correlation was observed with LV GLS of any layer. Unlike the correlation between LV GLS-endo and PWV, log aldosterone did not show a significant correlation with LV GCS-endo in patients without LV hypertrophy (Fig. 2).

**Table 3:** Simple correlations

	PWV		Log Aldosterone	
	R	*P*-value	R	*P*-value
LV GLS				
Transmural	0.145	0.090	0.062	0.511
Endocardial	0.169	0.046	0.052	0.586
Epicardial	0.162	0.056	0.056	0.552
LV GRS	0.036	0.710	−0.168	0.123
LV GCS				
Transmural	−0.039	0.689	0.240	0.024
Endocardial	0.016	0.870	0.251	0.019
Epicardial	−0.021	0.832	0.078	0.477
LV Torsion, peak	0.026	0.786	−0.139	0.191
LA GLS	−0.250	0.003	0.018	0.854

*LV* left ventricle, *LA* left atrium, *GLS* global longitudinal strain, *GRS* global radial strain, *GCS* global circumferential strain

Multiple regression analysis revealed that LV GLS-endo (beta = 0.223, *p* = 0.031) and LA GLS (beta = −0.318, *p* = 0.002) were independently correlated with PWV, even after controlling for confounding factors such as age, gender, body mass index, presence of diabetes mellitus, LV mass index, LV ejection fraction, and use of renin aldosterone angiotensin (RAAS) blockers (Table 4).

**Table 4:** Determinants for myocardial mechanical function

	β	t	*p*-value
LV GLS endocardium (R^2^ = 0.327)			
Age	−0.170	−1.48	0.142
Female gender	−0.112	−1.22	0.225
Body mass index	0.046	0.49	0.626
Diabetes mellitus	0.062	0.66	0.511
Peripheral SBP	0.024	0.26	0.795
LV ejection fraction	−0.419	−4.44	<0.001
LV mass index	−0.001	−0.01	0.995
Use of RAAS blocker	0.158	1.76	0.082
PWV	0.223	2.19	0.031
LV GCS endocardium (R^2^ = 0.164)			
Age	0.190	1.21	0.231
Female gender	−0.130	−1.06	0.294
Body mass index	0.076	0.63	0.534
Diabetes mellitus	0.108	0.87	0.385
Peripheral SBP	0.004	0.04	0.970
LV ejection fraction	−0.293	−2.35	0.022
LV mass index	−0.015	−0.13	0.896
Use of RAAS blocker	−0.091	−0.76	0.448
Log Aldosterone	0.201	1.61	0.112
LA GLS (R^2^ = 0.361)			
Age	−0.118	−1.05	0.297
Female gender	−0.096	−1.06	0.292
Body mass index	−0.179	−1.94	0.055
Diabetes mellitus	−0.158	−1.70	0.092
Peripheral SBP	−0.044	−0.50	0.622
LV ejection fraction	0.218	2.35	0.021
LV mass index	−0.065	−0.74	0.461
Use of RAAS blocker	−0.318	−3.59	0.001
PWV	−0.311	−3.12	0.002

*SBP* systolic blood pressure, *LV* left ventricle, *RAAS* renin angiotensin aldosterone system, *PWV* pulse wave velocity

## Discussion

This study presents correlation of LA and LA mechanical function with increased arterial stiffness and neurohormone level in patients with early hypertension without clinically apparent target organ damage. The principle finding from our results is that LV GLS, especially endocardium, and LA GLS were dominantly affected by central arterial stiffness in early hypertensive patients without clinically apparent target organ damage.

Two-dimensional speckling analysis provides sensitive assessment of mechanical function of LV and LA. Recently, LV GLS gained clinical significance in patients with hypertensive heart disease. Lee et al. showed that LV GLS, especially that of the epicardial layer, was the only independent predictor of events [13]. Saito et al. showed that LV GLS had additive predictive value to clinical risk factors and concentric LV hypertrophy for predicting adverse cardiovascular outcomes in asymptomatic hypertensive patients [14]. The mechanism for impaired longitudinal strain in hypertension has been studied recently. In an animal study, when LV GLS was compared with transmural histology of myocardium, fibrosis was predominant in the subendocardial layer, and longitudinal deformation of the LV was affected mainly by subendocardial fibrosis, whereas circumferential and radial strain were preserved until the late phase of the experiment [15]. Investigators demonstrated propagation of fibrosis from the subendocardium to the mid layer of the myocardium as HTN persisted, which affected radial strain in the late course. This finding is consistent with our data. From our data, PWV correlated well with LV GLS-endo. The significance of the correlation between PWV and LV GLS-endo was significant even in patients without LV hypertrophy.

In addition, LA GLS showed independent correlation with PWV. Although current guidelines recommend assessment for presence of LV hypertrophy as a sign of target organ damage, increased LA size is strongly associated with the duration and level of systolic blood pressure [16]. Change of LA mechanical function and enlargement of LA can occur even before LV hypertrophy [17]. It is interesting that our data showed a significant correlation between LA GLS and PWV when most of patients did not have an enlarged LA, reflecting changes in mechanical function before structural adaptation. A previous study with a small number of patients showed reverse remodeling of LA with treatment of quinapril and independent correlation with improved central arterial stiffness [18]. Our finding suggests that LA GLS can serve as a sensitive surrogate marker for treatment response or risk stratification in early hypertensive patients who do not have enlarged LA or LV hypertrophy.

The renin-aldosterone system is reported to cause subendocardial fibrosis and stimulate hypertrophy of myocytes [19, 20]. In our study, log aldosterone showed significant correlation with LV GCS-endo and LV GCS-trans from simple correlation analysis, whereas no significant correlation was observed between LV GLS and log aldosterone. The correlation between LV GCS and log aldosterone was not significant in patients without LV hypertrophy. Although this finding cannot be fully explained due to the cross-sectional nature of our study, the data suggest that neurohormonal factors affect myocardium in a different manner from mechanical stress due to increased arterial stiffness. A previous animal study suggested that circumferential strain is affected by later stages of HTN, as progressive fibrosis affects the middle layer of the myocardium. In our study, the correlation between LV GCS-endo and log aldosterone failed to show significance in multivariate analysis. This might reflect the characteristics of our study cohort. Many of the patients in our study were in early stages of hypertension, and 61 (31%) had undetectable plasma aldosterone and renin. The correlation between neurohormone and LV mechanics should be studied in a large number of patients with more advanced hypertension.

**Fig. 3:**
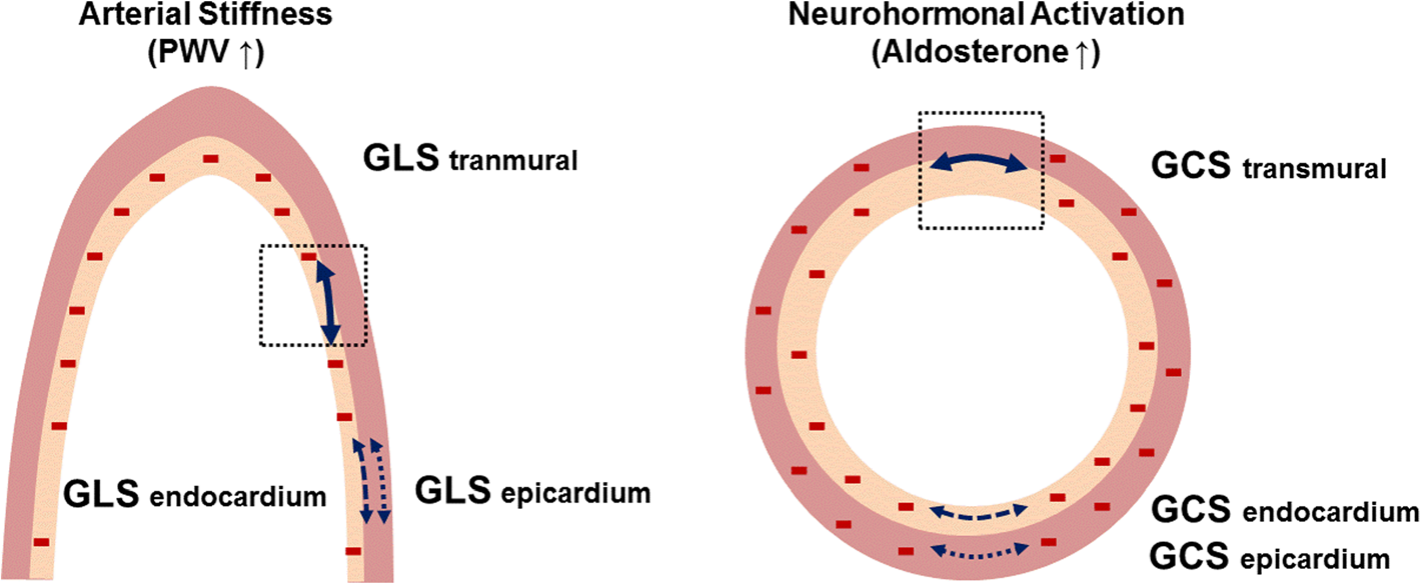
Hypothesis on LV and LA remodeling in early hypertension

Early detection of cardiac structural changes in early hypertension is important with regard to risk stratification. Based on the findings from our study, LV and LA remodeling in early hypertension can be hypothesized as outlined in Fig. 3. Mechanical stress from pressure overload impairs LV GLS of the endocardium and LA GLS. Neurohormones and aldosterone can impair LV GCS of the endocardium and transmural myocardium as hypertension progresses.

### Limitations

Several limitations of this study need to be noted. First, we included patients with early hypertension. Patients with LV hypertrophy represented only 27% of our cohort, and the small number of patients with LV hypertrophy might have affected the correlation analysis of the LV hypertrophy subgroup. Second, 51% of our patients were on RAAS blockers, which might have affected reverse remodeling of LV. However, we included use of RAAS blocker in multivariate analysis to minimize confounding effects. Third, because of the cross-sectional design of our study, we could not determine whether treatment of HTN can reverse abnormal LV mechanics. All of these issues are relevant and should be examined in future studies.

## Conclusion

Central arterial stiffness was independently correlated with LV GLS, especially endocardium, because the endocardium is the most susceptible to pressure overload among the three myocardial layers. Also, central arterial stiffness showed an independent negative correlation with LA GLS. Aldosterone showed significant positive correlation with LV GCS, especially endocardium from simple correlation, however, failed to remain significant in multivariate analysis. Different correlations between strain parameters and central arterial stiffness or aldosterone suggest different functional adaptations of LV and LA to arterial stiffness and neurohormone. In addition, two-dimensional layer-specific speckle-tracking echocardiography sensitively detected LV and LA mechanical adaptation in early hypertension, even without LV hypertrophy, and provided pathophysiologic insights into LV and LA remodeling in association with central stiffness.

## Abbreviations

AIx: Augmentation index
BP: Blood pressure
DBP: Diastolic blood pressure
GCS: Global circumferential strain
GLS: Global longitudinal strain
GLS-endo: Endocardial global circumferential strain
GLS-endo: Endocardial global longitudinal strain
GLS-epi: Epicardial global circumferential strain
GLS-epi: Epicardial global longitudinal strain
GLS-trans: Transmyocardial global circumferential strain
GLS-trans: Transmyocardial global longitudinal strain
HTN: Hypertension
LA: Left atrium
LV: Left ventricle
PP: Pulse pressure
PWV: Pulse wave velocity
RAAS: Renin angiotensin aldosterone system
SBP: Systolic blood pressure
SD: Standard deviation
